# United States Veterinary Medical Association

**Published:** 1891-07

**Authors:** W. Horace Hobkins

**Affiliations:** Secretary


### UNITED STATES VETERINARY MEDICAL ASSOCIATION.

The members of the U. S. V. Medical Association are hereby notified that the Trunk Line Association—Southern Pass Association have granted the usual one and one-third rates to all members of the profession attending the annual convention at Washington, September 15th to 17th, 1891.

This promises to be one of the most important and interesting meetings ever held by the association. The reports of the Committees on Food Inspection and Diseases will be given a more thorough hearing and discussion at this meeting than has been accorded them for many years. The relation of these subjects to the living world at this time in our nation's history and to sanitary science the world over, commands the presence of every member of the association, and a generous welcome will be accorded to every member of the profession who may attend.

Dr. C. C. Lyford, of Minnesota, will read a paper on the subject of "Barren Mares," a topic of interest to all. Dr. A. H. Baker, of Chicago, will read a paper. Other papers of much general interest are promised for this meeting, which will afford all an opportunity of being interested and add to the more thorough amalgamation of the profession of our entire country, so auspiciously begun at Chicago.

W. Horace Hobkins, *Secretary*.

